# A Comprehensive Review on Plant-Derived Mucilage: Characterization, Functional Properties, Applications, and Its Utilization for Nanocarrier Fabrication

**DOI:** 10.3390/polym13071066

**Published:** 2021-03-28

**Authors:** Mansuri M. Tosif, Agnieszka Najda, Aarti Bains, Ravinder Kaushik, Sanju Bala Dhull, Prince Chawla, Magdalena Walasek-Janusz

**Affiliations:** 1 Department of Food Technology and Nutrition, Lovely Professional University, Phagwara, Punjab 144411, India; tosifmansuri444@gmail.com; 2 Department of Vegetable Crops and Medicinal Plants, University of Life Sciences in Lublin, 20-280 Lublin, Poland; magdalena.walasek@up.lublin.pl; 3 Department of Biotechnology, Chandigarh Group of Colleges Landran, Mohali, Punjab 140307, India; aarti05888@gmail.com; 4 Department of Food Technology, School of Health Sciences, University of Petroleum and Energy Studies, Dehradun, Uttarakhand 248007, India; ravinderfoodtech2007@rediffmail.com; 5 Department of Food Science and Technology, Chaudhary Devi Lal University, Sirsa, Haryana 125055, India; sanjudhull@gmail.com

**Keywords:** nanohydrogel, food applications, biopolymers, polysaccharide

## Abstract

Easily sourced mucus from various plant parts is an odorless, colorless and tasteless substance with emerging commercial potential in agriculture, food, cosmetics and pharmaceuticals due to its non-toxic and biodegradable properties. It has been found that plant-derived mucilage can be used as a natural thickener or emulsifier and an alternative to synthetic polymers and additives. Because it is an invisible barrier that separates the surface from the surrounding atmosphere, it is used as edible coatings to extend the shelf life of fresh vegetables and fruits as well as many food products. In addition to its functional properties, mucilage can also be used for the production of nanocarriers. In this review, we focus on mucus extraction methods and its use as a natural preservative for fresh produce. We detailed the key properties related to the extraction and preservation of food, the mechanism of the effect of mucus on the sensory properties of products, coating methods when using mucus and its recipe for preserving fruit and vegetables. Understanding the ecological, economic and scientific factors of production and the efficiency of mucus as a multi-directional agent will open up its practical application in many industries.

## 1. Introduction

Plant-derived polymers have attained high demand in food and other industries due to their diverse industrial applications such as film coating, emulsifier, binder, and gelling agents, therefore they are excessively used in the textile industry, paper industry, and cosmetic industry [1,2]. Nowadays, due to the hazardous effect of synthetic polymers on human health, people showed major interest in plant-based naturally derived biopolymers (gums, mucilage, cellulose, and glucans) as an effective ingredient for the formulation of eco-friendly, sustainable, cost-effective products [3]. Moreover, a large number of polysaccharides can also be biosynthetically fabricated by several living organisms including plants, algae, animals, bacteria, and fungi [4]. Also, natural polysaccharides are used in the food industry as they are regarded as safe for human consumption [5]. Among various polysaccharides, plant-originated mucilage is widely used in various food industries due to its valuable broad-spectrum applications [6]. Generally, mucilage can be obtained from several plants or their different parts such as *Aloe vera*, *Salvia hispanica* seeds, *Cordia dichotoma*, *Basella alba, Plantago psyllium, Cyamopsis tetragonoloba*, *Cactaceae, Abelmoschus esculentus, Trigonella foenum-graecum*, *Moringa Oleifera,* and *Linum usitatissimum.* Plant-derived mucilage, due to its distinctive health (anticancer, angiotensin-converting enzyme inhibition extends to diabetes, and immunity stimulation) and food properties, is widely used as an active ingredient for the formulation of pharmaceutics, functional, and nutraceutical products [7]. Structurally, mucilage (a complex of polymeric polysaccharide) is mainly composed of carbohydrates with highly branched structures that consist of monomer units of L-arabinose, D-xylose, D-galactose, L-rhamnose, and galacturonic acid. They also contain glycoproteins and different bioactive components such as tannins, alkaloids, and steroids [8,9,10]. Also, mucilage produces an indefinite number of monosaccharides on hydrolysis, depending on the type of hydrolysis products obtained due to the nature of the polysaccharide. It can also further classify into pentose sugars (xylan) and hexose sugars (cellulose and starch) and can be considered as gum like components due to their similar physiological properties. However, both mucilage and gum are mostly related to hemicelluloses in composition, except the sugars produced by hemicelluloses such as xylose, glucose, and mannose instead of sugars produced by the gums such as galactose and arabinose [11,12]. Moreover, that can be utilized in several applications such as edible coating, wound healing, tablet formation, encapsulation, water purification, and various nanocarriers. Mucilage exhibits an excellent functional property, however, due to the hydrogen bonding in between different functional and other polar groups, they also have an important role in film, emulsion, coated metal nanoparticles, and gel formation [13]. In recent years, nanostructured hydrogels and mucilage coated metal nanoparticles are intensively used as a significant delivery vehicle for various hydrophilic and hydrophobic components [14]. For the formulation of nanohydrogel, different types of biopolymers and cross-linking polymers can be used and mucilage can act as either a primary biopolymer or a cross-linking component for the formulation of nanohydrogel [15]. Several reports have been published on the formulation of stable nanohydrogels using mucilage as an active component and researchers revealed various therapeutic and food applications of the formulated nanohydrogels [11,12,13,14,15,16,17]. Furthermore, nanohydrogels formulated with mucilage exhibit higher stability than that of other conventional plant-based biopolymers. Furthermore, metal nanoparticles coated with polymeric carbohydrates such as starch, dextran, chitosan, and mucilage are the most abundant nanocarriers used for targeted drug delivery. Because, in addition to increasing blood circulation time by hiding them from the immune system, their polymeric shells enable them to transfer and release the drug during biodegradation [16,17]. However, only a few reports are published on the comprehensive knowledge of plant-derived mucilage, therefore, the present review is emphasized on the physicochemical properties, characterization, health, and functional attributes of the mucilage. Also, the application of mucilage crosslinked nanohydrogels and mucilage coated metal nanoparticles are discussed with mechanisms and schematic diagrams.

## 2. Origin of Mucilage in Different Plant Parts

The Mucilage is a water-soluble edible adhesive material that constitutes carbohydrates and uranic acids units present in different parts of plants including the mucous epidermis of the outer layer of seeds, bark, leaves, and buds [18]. The majority of plants produce mucilage from the seed coat and this process of producing mucilage is termed Myxospermy and some plant species produce it from the fruit epicarp which is known as Myxocarpy. Plants producing mucilage from seed coat belong to the family *Plantaginaceae, Acanthaceae, Linaceae,* and *Brassicaceae*, while Myxocarpy (fruit mucilage) is commonly found in families like *Poaceae, Asteraceae*, and *Lamiaceae* [19]. The presence of mucilage on the seed coat prevents the plant from early seedling development and drought stress during the germination. depending upon its origin it is characterized into many groups including hair secretion, intracellular mucilage, and cell membrane mucilage [11]. The mucilage obtained from the seed coat is classified into three classes that are endosperm non-starch polysaccharide (galactomannans), cell wall material of the endosperm (soybean hemicelluloses and xyloglucans), and mucilaginous constituents of the seed coat (flaxseed, Chia seed, and yellow mustard) [20,21]. Mucilage develops a jelly-like structure around fruit and maintains moisture and prevents seeds from completely drying out and therefore act as a hydrating agent and also acts as an energy reservoir [22,23]. Mucilage also plays a significant role in the control of germination, the promotion of dispersal, and soil adhesion, root mucilage is usually exhibited from the outer layers of the root cap, consisting of mostly root border cells and polysaccharides, which produce various chemical substances such as flavonoids, phenolics acids, amino acids, galactosidase, antibiotics, sugars, peroxidase, proteins, and anthocyanins [24]. Moreover, root mucilage plays a very important role for plant growth, such as for the maintenance of root-to-soil touch, root tip lubrication, soil microaggregate stabilization, water storage ability, selective storage, and the absorption of ions (Na^+^, Cd^2+^, Pb^2+^, and Al^3+^) through root cells. Furthermore, it is primarily secreted by the secretory vesicles of hypersecretory root cap cells as a coagulated polysaccharide (poly-galacturonic acid) and is subsequently passed during root extension to older root areas, but epidermal cells are also effective in secreting mucilage [25,26,27]. Mucilage is also produced in the leaves and buds of several plant species; it may allow the leaves to retain water capacity when soil water deficits emerge; therefore, it helps in the storage of food and water. Various mucilage containing plants and their origins are highlighted in Table 1.

## 3. Extraction of Plant-Derived Mucilage

**Figure 1:**
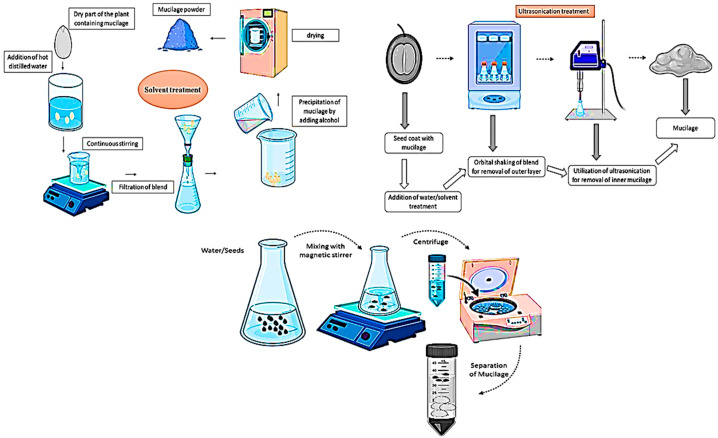
Extraction of mucilage from plant parts using solvent extraction, microwave assisted, and centrifugation process.

Mucilage can be extracted from any part of the plant is considered a valuable natural polysaccharides source with excellent potential in pharmaceutical and food applications. several studies said that the yield, functional, and rheological properties of mucilage are highly dependent on the extraction method and extraction conditions [28]. Generally, all the extraction method of mucilage comprises of two successive procedures which are maceration and precipitation. Usually, the maceration extraction method of mucilage is simple and valuable, although the disadvantage of maceration is low efficiency and long extraction time [33]. This method consists of soaking the raw material in the chosen solvent at room temperature with regular agitation. maceration for extraction of mucilage is typically done using the low ratio of solid-liquid and hot water treatment. acid solutions, ammonium oxalate, and EDTA are also used to improve mucilage extraction [26]. According to several studies, the water-extracted mucilage showed high viscosity than alkali and acid-extracted mucilage due to marked differences in the mucilage structure of monosaccharides. However, the limitations of these methods are higher levels of protein with low yields of mucilage and subsequent denaturation. Apart from its nutritional value, protein is known as an impurity in the final product which affects its purity and restricts its industrial use due to the instability and undesirable taste caused by microbial spoilage. while protein presence in the mucilage has been identified regardless of the extraction methods used and an emulsifying action has been taken to demonstrate that protein is desirable to improve the texture and consistency of emulsions and beverages [34]. The general outline revealed an increase in protein levels as time and temperature of maceration increased, resulting in a higher molecular weight of mucilage. Also, higher maceration temperature and extended stirring period contributed to highly colored mucilage, which is unacceptable for industrial usage. Acid pre-treatment should be considered in order to protect against this color effect [35]. Solvent treatment is a conventional extraction technique of mucilage. generally, the aqueous procedure included the mucilage extracted from the dry parts of the plant (seeds, leaves, roots, stems) by using hot distilled water. The procedure occurs under continuous shaking and stirring of solution. The solution is then filtered, these stages can be repeated often. Consequently, the mucilage is precipitated by the addition of alcohol to the filtrate. Then, precipitated mucilage is dried via freeze-drying or in an oven to obtaining the final mucilage powder [29] whereas ultrasonication treatment is a emerging non-thermal technique for mucilage extraction with several applications in the pharmaceutical and food industry. Furthermore, in some plant parts, two layers of mucilage (Arabidopsis seed coating) make the extraction process very difficult; therefore, ultrasonication treatment can be much helpful in such cases. The outer layer of the seed coat can be extracted through shaking, and an inner layer of the seed coat is composed of rhamnogalacturonan I, which is a very difficult challenge to extract. in this case, ultrasonic treatment for 20 s showed excellent results. ultrasound with low-frequency has been used for mucilage extraction by disrupting the biological cell wall through the formation of pores. The formation of cavitation bubbles and their resulting collapse produce high spots with higher pressure and temperature, capable of breaking the bonds between the mucilage and seed coat. As a result, the amplitude of the ultrasounds, extraction temperature, and time must all be selected carefully to prevent the extraction of undesirable compounds and mechanical disturbance of the seeds [36]. Likewise, microwave is a thermal emerging extraction method that can be used for the extraction of mucilage from several parts of the plant. Moreover, microwave-assisted extraction is a potential alternative method providing advantages in terms of improved extraction efficiency, reduced solvent consumption, and time reduction. Conventional aqueous extraction methods include the effects of solvent, pH, and temperature, which change the nutritional value, functional and structural property of mucilage [37]. Using hot water extraction with a long extraction period is considered to be cost-effective, although it can reduce the consistency of the mucilage. Several studies said that conventional hot water extraction methods result in the loss of heat liable compounds of mucilage, to overcome this major drawback, microwave-assisted extraction with extraction at 300 to 400 W for 120 to180 s was used, resulting in a high mucilage yield. Consequently, enzymes also play a very crucial role in the extraction process due to their wide range of applications in the food industry. During the enzymatic hydrolysis for reduction of viscosity and molecular weight of mucilage, several enzymes can be used such as rhamnase, arabinase, xylanse, and mannonase [13]. Furthermore, cold extraction can be applied in order to produce more viscous mucilage, which is more natural but with a lower yield compared to the hot extraction method [38]. Moreover, an increase in the ratio of solid-liquid is proportional to more yield due to more availability of solvents, which improves the driving force of mucilage from raw plant material. However, the high ratio of solid-liquid cannot significantly affect mucilage yield, also consisting of high process costs. In this context, Elboutachfaiti et al. [37] extracted mucilage from flaxseed mucilage after maceration in hot water, six rhamnogalacturonan fractions of flaxseed mucilage were extracted and purified by using ion-exchange chromatography. As regards the pH effect on the extraction yield of mucilage, a significant increase in the yield of mucilage was observed with an increase in pH due to the separation of the acidic groups such as uranic acids, and due to the attraction between the negatively charged ones, which increased the solubility of the mucilage, although further decreased after a certain pH value. Moreover, lower pH values of mucilage are likely to improve protein recovery due to protein solubilization. Although, below 3 pH, the action of acid can result in a lower protein yield due to its hydrolysis [39]. In order to obtain the quality polysaccharides (mucilage) and highest yield, it is necessary to improve the extraction procedure (Figure 1). Deionized water can also be used for the extraction of mucilage [39,40].

## 4. Structural Chemistry of Mucilage

**Figure 2:**
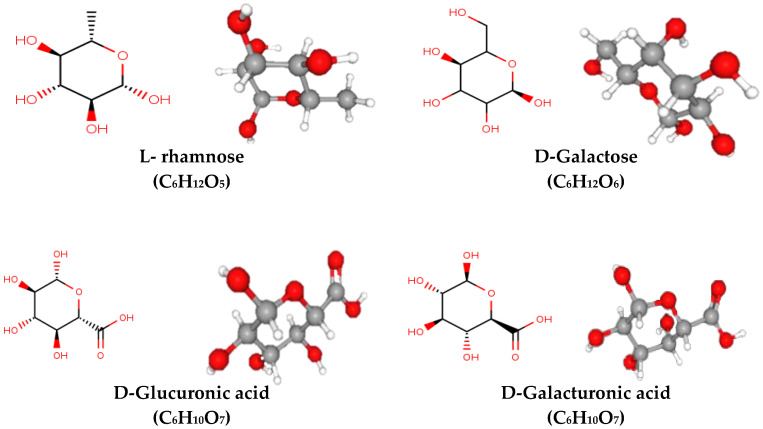
Chemical structure of different mucilage (L- rhamnose (C_6_H_12_O_5_) 6-deoxy-hexose or methyl-pentose, D-Galactose (C_6_H_12_O_6_) C-4 epimer of glucose, D-Glucuronic acid (C_6_H_10_O_7_) sixth carbon atom oxidized to a carboxylic acid, D-Glucuronic acid (C_6_H_10_O_7_) an aldehyde group at C1 and a carboxylic acid group at C6).

The mucilage is a water-soluble component constituting different functional chemical components with potential human health benefits [41]. Mucilage and gum are a subgroup of hydrocolloids containing monosaccharides linked with organic acids and are close to each other due to the hydrophilic and hydrocolloid components that create a sticky solution or gel in the presence of water [2]. Plant hydrocolloids (Gum and mucilage) contain pentose, galactose and methyl pentose sugar joined to uranic acid residues by glycosidic linkages. The terminal atom of carbon (at the opposite side of the carbonyl chain) of the monosaccharide unit may occur in an oxidized (carboxylic acid) form. Six carbon with aldohexose in the form of a carboxylic acid is termed uranic acid [24]. Furthermore, Monosaccharides are the most common carbohydrate molecules that cannot be broken down into simpler sugar molecules by hydrolysis and are occasionally referred to as simple sugars. Mucilage present in plant consists of two main polysaccharides pectin and hemicellulose each of which is comprised of rhamnogalacturonan, and arabinoxylans respectively [42]. Generally, mucilage arabinoxylans encompass by *β*-1,4-linked xylose backbones, they are mostly replaced by 1-3 sugar residues at O-2 or/and O-3 positions. While, mucilage rhamnogalacturonan I (RG-I) comprise a backbone of the repeating disaccharide of ***α***-(1,2)-rhamnose and ***α***-d-(1,4)-galacturonic acid [43]. Moreover, there is some evidence that rhamnogalacturonan I side chains can be covalently bound to hemicelluloses, creating a super-macromolecular polymeric network. The neutral sugars are mainly D-xylose, L-arabinose, and D-galactose, with the proportions and types of neutral sugars varying with the origin of mucilage. In mucilage, both carboxyl and hydroxyl groups are present as functional groups due to the presence of these functional groups it acts as a polyelectrolyte [44]. The different chemical structures of polysaccharides are illustrated in Figure 2.

In a study done by Hosseini-Parvar et al. [45], Beikzadeh et al. [9], Timilsena et al. [46], and Mirhosseini et al. [47] observed that basil seed mucilage contains xylan (24.29%), glucan (2.31%), and glucomannan (43%), chia seed contains glucose (19.6%), galactose (6.1%), arabinose (9.6%), xylose (38.5%), galacturonic acid (5.3%), and glucuronic acid (18.7%), cress seed mucilage contains glucose (1%), fructose (6.8%), arabinose (19.4%) rhamnose (1.9%), glucuronic acid (6.7%), and galactose (4.7%), and flaxseed contains xylose (21.1–37.4%), fructose (5–7.1%), galactose (20–28%), and arabinose (9.2–13.5%) [17]. All these studies conclude that mucilage present in the seeds coat of plants of different species exhibits different forms of carbohydrates and uranic acid units.

## 5. Characterization of the Mucilage

This technique is used to determine the chain configuration, i.e., the microstructure of the polymer when present in either a solid state or a liquid state. This technique can also be applied to any form of the sample containing a spin-filled nucleus. In this context, Singh et al. [2] observed nuclear magnetic resonance spectra (^1^H and ^13^C) of the mucilage of the seed/fruit of *Diospyros melonoxylon Roxb* at 400 MHz, and revealed that many sugar compositions are consist of CH and OH groups of mannose (δ 3065 to δ 3060 ppm), CH group of rhamnose (δ 72.2 ppm), CH group of arabinose (δ 70.1–δ 71.8 ppm), the CH_2_ group of arabinose (δ 3.81 to δ 3.55 ppm), and the CH group of mannose (δ 72.3 ppm), respectively. Likewise, Deore et al. [48] and Dehghani et al. [49] studied the composition of the mucilage of *Cassia obtusifolia* and chia seeds and concluded that the mucilage of *Cassia obtusifolia* contains the CH group of arabinose (δ 69.61–δ 71.25 ppm), glucose (δ 4.15 and δ 3.84 ppm), OH and CH groups of mannose (d 3.62 and d 3.41 ppm), methyl group (1.23 ppm), non-anomeric protons (3.1 and 4.1 ppm), OH and CH_2_ groups of arabinose (δ 3.55 and δ 3.39 ppm), while chia seed mucilage contains OH and CH groups of mannose (3.6 and 3.65 ppm), OH and CH groups of arabinose (3.55 and 3.81 ppm), and the bond between methyl and protons with C_6_ and C_4_ of galactose. In another study done by Devi et al. [50], it was observed that flaxseed mucilage contains methylene and thio portions of thioglycolate resonance at the peak value of 4.14 and 5.56 ppm. FTIR analysis is performed to determine chemical structure and functional groups of mucilage, FTIR spectroscopy can be used in wavelength regions between 4000 and 400 cm^−1^ at a resolution of 2 cm^−1^ or 4 cm^−1^. The area between 800 and 1200 cm^−1^ is known as the carbohydrate fingerprinting area. Mucilage contains polymers such as CH_2_, O-H, C-H, C-O-C, and the carboxylate group was observed in different studies. It was also observed that the spectra obtained from mucilage showed a huge peak value at the range of 3500–3300 cm^−1^, the absorption band at around 3000–2800 cm^−1^, 1270–1080 cm^−1^, at 1600 cm^−1^, and 1400 cm^−1^ confirming the vibrational stretching of the polymeric O-H group, CH_2_ and C-H group, C-O group, Carboxylate asymmetric stretching, and symmetric stretching respectively [51,52,53]. Naji-Tabasi et al. [54] conducted a study on basil seed mucilage, the FTIR analysis of the study indicated the existence of uronic acids, absorptions at wavelengths 1600 and 1400 cm^−1^ confirmed the presence of uronic acid assigned to C-OO asymmetrical and symmetrical stretching, respectively. Consequently, Pratik and Shadique [55] extracted mucilage from the fruits of Tilkor (*Mamoradica monadelpha*), the mucilage sample was prepared in powder form by using freeze-drying. FTIR result confirmed the presence of complex carbohydrate (starch), moreover, FTIR of Tilkor mucilage showed the vibrational stretching of C-H bending of Alkynes, C-H bending in aromatic rings, C-C Stretching vibrations, free O-H groups Vibrations, and C=N bond showed Aminoacids/ proteins and the absorption band wavelength at around 685 cm^−1^–665 cm^−1^, 900 cm^−1^–625cm^−1^, 822 cm^−1^, 3710–3513 cm^−1^, and 1623 cm^−1^ respectively.

Rheological characterization is used to determine the shear rate-dependent flow behavior of the mucilage solutions, and is generally examined over a shear rate range of 0 to 100 Hz. The rheological characterization of the mucilage of seeds from different plants was performed by several scientists. In this context, Punia et al. [56] measured the rheological characteristics of the mucilage of chia seeds by an oscillatory shear (range of frequency stress sweep is about 1 Hz to 10 Hz) and revealed the highest correlation coefficient of chia seed (R^2^ > 98.58) and with the increase in shear rate, they observed a rapid decrease in the viscosity of the solution. In another study by Capitain et al. [57] it was observed that the chia seed dispersions viscosity increases by increasing the concentration from a range of 0.25 to 1.00 (*w/v*). Likewise, Abbastabar et al. [58] characterized quince seed mucilage and observed curve turned from the linear range at strain 11.4%. Increases of storage modulus and decrease of viscoelastic linear range (7.7%) were observed in the presence of 0.2 M NaCl as NaCl shows its dual behavior on the mucilage or gum. Quince seed mucilage has high activation energy which is about 6988.74 J mol^1^. activation energy is usually used to determine the chain flexibility of biopolymers. Keshani-Dokht et al. [59] in their study observed a decrease in the magnitude of *Cordia myxa* mucilage and an increase in solution concentration from 0.99 to 0.89 and 0.2–2% respectively. In their study, they observed that the mucilage solution has a tendency of more shear thinning at a high concentration level. The activation energy of *Cordia myxa* mucilage was calculated at about 446.23 KJ. The thermogravimetry analysis (TGA) technique is used for measuring the mass variation in a particular mucilage-containing sample as a function of temperature in a stable atmosphere [53]. The vibration of mass can be negative when the part of the sample is transformed in vapor and that can be positive when the sample is subjected to corrosion or oxidation [60]. The thermal stability of mucilage can be measured in two conditions such as isothermal (temperature is kept constant) and dynamic (temperature is increased at a linear rate) [6]. Thermal stability and thermal behavior of *Manilkara zapota* (Linn.) Seed mucilage data showed that heating at 10 °C /min from 0 °C to a maximum of 900 °C resulted in two mass loss events according to the derivative thermograms and primary thermograms. Furthermore, there was 41.17% weight loss at 178.6–359.7 °C temperature range in the first Decomposition stage and 30.06% weight loss at 359.7–600.6 °C in the second Decomposition stage. Besides, at the same temperature range the enthalpy (315.8729 and 3624.787 J/g), DTG peak (350.3 °C and 614.4 °C), and heat change (138.4354 and 1082.215 μVs/mg) were observed for both first and second decomposition stages [61]. Differential scanning calorimetry (DSC) has emerged as an excellent physical technique to investigated physical and chemical or exothermic and endothermic changes that occur in the mucilage during thermal processing [62]. The high thermo-stable property of mucilage can be utilized in paints for stabilizing suspensions or emulsions, improve bake-stability in cakes, preventing crystal growth, and improving freeze-thaw stability [6]. The outcome of the DSC analysis of *Diospyros melonoxylon* Roxb seed mucilage discovered the glass transition temperature is 78 °C. the high intense peak was observed in Differential scanning calorimetry thermograms at around 200 °C which was an endothermic transition. The large endothermic peak correlates to the hydrophilic nature of the functional groups of chia seed mucilage and can be due to in regular packing structure of the gums [63]. The endothermic peak transition temperatures (Te, To, Tp) of chia seed mucilage were 215 °C, 52.8 °C, and 107.9 °C, respectively. 233.9 J/g was enthalpy change value of chia seed mucilage. In the case of exothermic peak transition temperatures (Te, To, Tp) of chia seed mucilage were 354.9 °C, 277.7 °C, and 316.8 °C, respectively, and the enthalpy value of exothermic peak (101.9 J/g) was lower than endothermic. The high enthalpy value of mucilage indicates high energy needed in releasing water-related with the loss of crystallization as well as hydrogel bounded [56].

## 6. Functional Properties of Mucilage

Hydrocolloid polymers in the food industry and pharmaceutical industry are generally made up of large molecular biopolymers. A biopolymer contains a hydroxyl group which leading to viscous dispersions, increases the water attraction. Also, those hydrocolloids are widely used as a stabilizer, thickener, dietary fiber, whipping agent, and fat replacer. Moreover, they also have applications in crystallization inhibition, edible coatings, edible films, and encapsulating flavors [13,64]. The different functional properties of mucilage are shown in Table 2.

### 6.1. Water Holding Capacity of Mucilage

All the scientific literature has shown that high water holding capacity is due to the presence of hydroxyl groups and protein substituents in the gum and mucilage structure. In 1996, the Food and Agriculture Organization of the united nation (FAO) described it as a potential source of polysaccharides due to its excellent property in aqueous solutions at low concentrations [63]. Water holding capacity is the capacity of a moist polymer or sample to hold water when exposed to an external centrifugal gravity force or compression. It comprises the sum of linked water, physically trapped water, and hydrodynamic water, the latter of which contributes most to this capacity [13]. The low amount of water holding capacity can be attributed to the high solubility of mucilage even at a high concentration which leads to an inability to form a gel. Furthermore, mucilage is widely selected in the food system (fruits, vegetables, poultry, and fish) and contributed to improved emulsifying and water binding capacity of food [60]. According to a study the water holding capacity of *Cordia myxa* mucilage (14.94 ± 2.44 g/g), *Opuntia dilleniid* mucilage (4.00 ± 0.10 g/g), sweet basil seed mucilage (97.5 ± 2.4 g/g), Tamarind seed at 25 °C (0.18 ± 0.014 g/g), at 45 °C (0.22 ± 0.167 g/g), and at 65 °C (1.07 ± 0.025 g/g), which is shown that the water holding capacity of tamarind seed is increasing as increasing of temperature [13,26,59,71,72].

### 6.2. Oil Holding Capacity of Mucilage

Oil holding capacity is the most important functional property of hydrocolloids, which indicates oil absorption capacity. According to the reports, due to an increase in the accessibility of nonpolar chains with increasing molecular mobility of hydrophobic hydrocarbon domains, plant-derived mucilage show increased oil binding capacity with increases in temperature. The oil holding capacity of tamarind seed mucilage at 25 °C (0.068 ± 0.014 g/g), at 45 °C (0.104 ± 0.010 g/g), at 65 °C (0.133 ± 0.004 g/g); chia seed mucilage (12.97 ± 1.90 g/g); guar gum (0.87 ± 0.06 g/g); and basil seed mucilage (8.37 ± 1.02 g/g) [13,26,27,73,74]. Good oil holding values indicate that mucilage could improve the texture of food products. Fruit or seed extracted mucilage has good oil binding capacity due to presence of monopolar molecules, therefore, it can trap higher amounts of oil particles and also prevent oil and the loss of flavor from food systems. Therefore, fruit or seed-derived mucilage can be used as a good functional ingredient in formulated foods [75].

### 6.3. Emulsifying Property of Mucilage

Plant-derived polysaccharides such as gum, mucilage, and starch as well as Carboxymethyl cellulose (CMC) are widely used in emulsion products [76,77]. Polysaccharide mucilage and gum are attracting attention towards commercial use in the pharmaceutical and food industry due to their excellent emulsifying properties, where they are used for suspension of particulates, stabilization of emulsions, control of crystallization, the thickening, and formation of films, encapsulation [30]. The emulsion products prepared from different plants are checked for their stability by several scientists to use them for different purposes in different food and pharmaceutical industries. Sangeethapriya and Siddhuraju [78] prepared the emulsion products from quince seed mucilage and they observed that the stability of the resulting product was 94.89% likewise Behrouzian et al. [79] observed the high emulsifying capacity (92%) of cress seed mucilage while in another study by Jouki et al. [80] it was observed that emulsifying capacity of *Zizyphus mauritiana* fruit mucilage is about 54.12% and emulsifying stability is 42.14%.

### 6.4. Foaming Property of Mucilage

Good foaming property is associated with the elastic structure of the mucilage, foaming property of mucilage is depending upon many factors, such as the presence of further compounds in the hydrocolloid, molecular weight, protein, structure, and carbohydrates. The good foaming capacity of mucilage is highly associated with the flexible structure of mucilage that can reduce the surface tension [81]. The foaming capacity and foaming stability of *Abelmoschus esculentus* mucilage is 50–51% to 50–62% and 36.04%, 1% *w/v* respectively, foaming capacity flaxseed mucilage reported about 75%, and Plantago seed mucilage has foaming stability (88.4%) [13,66,82].

### 6.5. Antioxidant Property of Mucilage

Plant-derived polymers contain different phenolic compounds including flavonoids and polyphenols as bioactive compounds. These bioactive compounds prevent the chain propagation reactions originated by free radical reactions and prevent disease-related oxidative damage [83,84,85,86]. Sangeethapriya and Siddhuraju [78] studied the antioxidant activity of the polyphenols isolated from *Zizyphus mauritiana* fruit mucilage against DPPH- 2,2-diphenyl-1-picrylhydrazyl is 5.27 g mucilage/g DPPH, superoxide is 85.12%, ABTS- 2,2-azino-bis-3-ethylbenzothiazoline-6-sulphonic acid is 16587.32 mmol Trolox Eq./g, and hydroxyl is 76.13%. Sardarodiyan et al. [87] also studied the antioxidant activity and total phenolic compounds of *Lallemantia royleana* seed mucilage and in their study, they observed antioxidant activity of 528.54 μg/mL of DPPH and total phenolic content of 82.56 ± 1.6 μg/GAE. In two different studies done by Jouki et al. [80] and Silveira Coelho et al. [88] the antioxidant property of Quince seed and phenolic content of chia seeds were 29.88% and 641.71 μg/GAE, respectively.

### 6.6. Antimicrobial Activity of Mucilage

Nowadays, food safety and food quality are major challenges for all countries, it is estimated that there are around 30% of people are suffering annually from food illness disease in developed countries [84,85]. Many natural polymers such as proteins and carbohydrates have antibacterial and antifungal properties, which prevent or reduce the growth of microorganisms in food. Infectious microorganisms are extensively spread everywhere on the earth which causes a serious problem for society. Furthermore, natural polymers are more effective in the treatment of those diseases and also prevent several side effects that can be led by antimicrobial agents [86]. While mucilage has an antibacterial and antifungal property which can also prevent or reduce the risk of food-borne illness disease and food spoilage. Much seed-based mucilage has an antimicrobial property such as Chia seed [86], Cress seed [78], okra seed [65], and quince seed [80] Tantiwatcharothai and Prachayawarakorn [89] prepared Basil seed-based sponges hydrogel for measuring the antibacterial activity (against *S. aureus* and *E. coli*) in wound dressing. ZnO nanocomposite and borax crosslinking were also incorporated with basil seed mucilage. The result showed that there was no effect of borax in the antibacterial activity of Basil seed mucilage sponge. Whereas, ZnO nanocomposite loaded resulted in good antimicrobial activity against the gram-positive (*Staphylococcus aureus)* and gram-negative *(Escherichia coli)* bacteria. Furthermore, better antibacterial activity was observed against gram-positive (*S*. *aureus)* than that of gram-negative *(Escherichia coli)* because of the structural difference of the cell wall. Gram-negative bacteria possess a complex cell wall, while gram-positive bacteria contain a thick cell wall of peptidoglycan, therefore, it is more resistant to attack by the microbial agent. Jouki et al. [80] prepared quince seed mucilage-based film with the incorporation of thyme essential oil in different concentrations ranging from 0 to 2.0%. The film was separated against eleven food associated with bacterial strains by agar disc-diffusion assay. And observed that 1% thyme essential oil containing film was more effective against all microorganisms including gram-positive and gram-negative bacteria such as *Staphylococcus aureus*, *Listeria monocytogenes,* and *Shewanella putrefaciens*. Mujtaba et al. [90] also prepared chia seed mucilage composite films with the incorporation of cellulose nanofibrils at various concentrations by using the solution casting method. Microorganisms such as *Escherichia coli, Pseudomonas aeruginosa, Staphylococcus mutans, Salmonella typhimurium, Bacillus thuringiensis,* and *Staphylococcus aureus* were incubated in LB agar at 37 °C for 24 h. the result indicated that chia seed-based cellulose nanofibrils composite film improved antimicrobial activity with the increasing of cellulose nanofibrils concentrations (3–6%) and observed that mucilage films containing 6% cellulose nanofibrils had the highest antimicrobial capacity against all the exanimated microorganisms. The inhibition zone range of all the exanimated microorganisms was *Escherichia coli* (20.17 mm), *Pseudomonas aeruginosa* (18.25 mm), *Staphylococcus mutans* (17.79 mm), *Salmonella typhimurium* (6.12 mm), *Bacillus thuringiensis* (17.19 mm), and *Staphylococcus aureus* (18.89 mm). The antimicrobial activity of different mucilage-containing plants is represented in Table 3.

**Figure 3:**
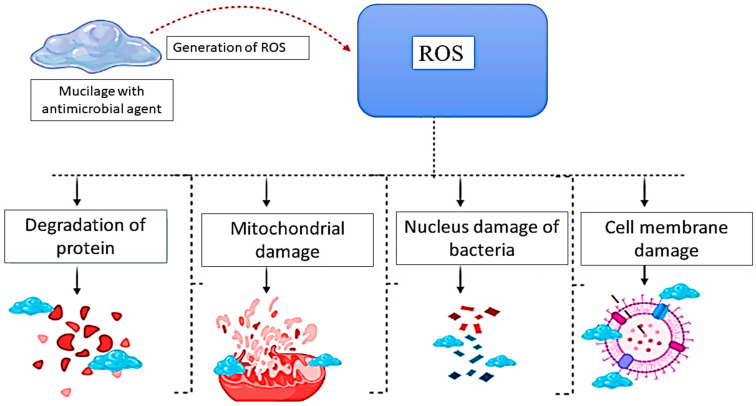
Possible mechanism of antimicrobial efficacy of plant-based mucilage.

Seed and fruit mucilage show excellent activity against pathogenic gram-positive and gram-negative bacteria. The mode of action of their mechanism is proposed by several authors in various theories as shown in Figure 3, mucilage with incorporated substance exhibit antibacterial ability by binding to bacterial membranes and enters inside the cell, thus resulting in the damage of the Nucleus: DNA damage, Degradation of Protein, Cell membrane damage and Mitochondrial damage [37].

The mucilage is helpful in the development of free radicals by the membrane, which leads to porosity and the death of the cell. Moreover, mucilage generates ROS (reactive oxygen species), which are free radicals that easily react with other molecules in the cell due to the presence of oxygen, which causes the damaging of proteins, RNA, and DNA [91]. Oxidative stress of ROS is a major cause of damage to the cell membrane due to lipid peroxidation. The mucilage can attach to the bacterial cell wall through several interactions in the cell membrane; therefore, the reducing the viability of the bacterial cell by inhibiting DNA replication. The bacterial cell comprises DNA bases enriched in sulfur and phosphorus, these biomaterials associate with the mucilage. Moreover, mucilage can break down the cell membrane permeability of pathogenic bacteria such as *E. coli*, *S*. *aureus*, *Bacillus* spp., and *Pseudomonas* spp., and also reduce the amount of ATP (adenosine triphosphate) by regulating the production of ATPase enzymes [92,93].

## 7. Application of Mucilage

The plant-derived additives or polymers due to their positive escape gaining importance in recent days and can be utilized as natural thickeners or emulsifiers for the human diet, therefore, act as an alternative to synthetic polymers or additives [19]. Moreover, mucilage derived from plants due to their property of elasticity can form a large number of networks molecules, therefore, can be widely used as an edible film or edible coating in food packaging applications in food industries, disintegrants in tablets, application of tablet binders, and several other applications in the pharmaceutical industry [11,94].

### 7.1. Mucilage as a Coating Material

Mucilage derived from plants can enhance the shelf life, storage capacity and also reduce the loss of moisture, therefore, extensively used as an edible coating in food packaging industries [95]. The mucilage has high tensile strength and barrier properties against different gases which reduce the firmness and weight loss of coated products [96]. Several studies have been conducted to check the effect of mucilage obtained from seeds of plants by different scientists [97]. In a study by Alizadeh Behbahani et al. [39] edible coating of Shahri Balangu seed mucilage (SBM) incorporated with cumin essential oil (CEO) on beef slices was done and a sensory study of beef slices during the storage period of 9 days was carried out. It was observed that mucilage coated beef slices reduced the loss of lipid oxidation, microbial counts (total viable count TVC), *Escherichia coli*, psychotropic count, yeast, and molds during the storage period. There was no adverse effect of SBM with CEO on sensory characteristics of beef and it conferred an excellent texture to the product as compared to the control sample. The different food applications of mucilage are shown in Table 4.

### 7.2. Application of Mucilage as Encapsulation Agents

Encapsulation of food ingredients, pharmaceutical ingredients, and nutraceutical ingredients is an effective technique to improve the stability of targeted compounds, apart from providing benefits on delivery characteristics [99]. The technique is used in the flavor industry to conceal undesirable taste/flavor notes such as the characteristic pungency and bitterness of polyphenols. This can be applied to a decline in the formation of rancidity in oil powders [84]. The preparation of the wall or filler material for the encapsulation of the bioactive substance is an important step because it influences the properties of the emulsion, compound preservation, and final product stabilization. several food-grade polymers have been discovered for nano and microencapsulation applications such as lipids, proteins, and carbohydrates. Furthermore, mucilage has a good ability to form a dense network after drying and their high molecular weight carbohydrate polymers make them suitable for nano and microencapsulation of food and bioactive compounds [102]. High molecular weights can aid in the retention of more key substances and increase stability. Plant-based mucilages are currently being investigated as wall materials for various encapsulation tests, in addition to their other applications.

### 7.3. Application of Mucilage in Wound Healing

**Figure 4:**
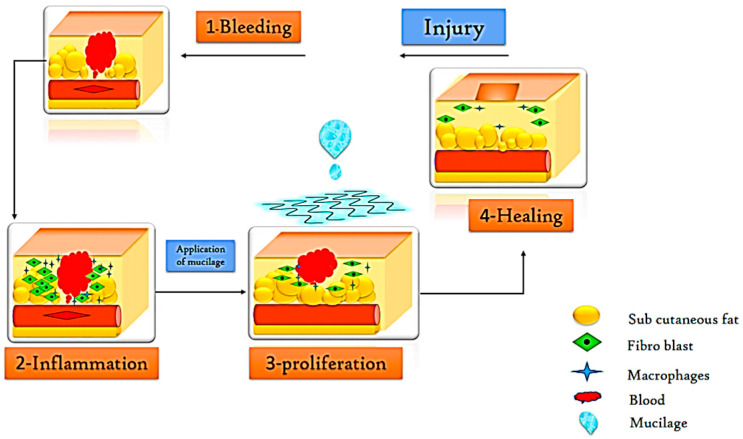
Application of plant-based mucilage in wound healing.

The skin comprises the three layers of the dermis, epidermis, and hypodermis, these are known as the largest organ in the body. A wound is a mechanical or physical injury that results in breaking or opening of the skin [104]. Dressing a wound to prevent it from bacterial infection is most important to allow the wound to heal quickly. Generally, dressing absorbs the exudates from the wounded skin and prevents the pathogenic microorganisms by producing moisture at the border of the wound [89]. Plant-derived polymer due to hydrogen bond-forming groups are more extensively used for advanced and traditional wound healing and drug delivery system [105]. Mucilage-based hydrogels are non-toxic, biocompatible, biodegradable, and easily available, therefore, used for the wound healing process. These hydrogels can be naturally removed or applied with wound beds without any significant interference [106]. Likewise, Tantiwatcharothai and Prachayawarakorn [89] studied the antimicrobial effect of basil seed mucilage-based hydrogel and they observed good antimicrobial activity against pathogenic bacteria *E. coli* and *S. aureus*, isolated from the wounds. The application of mucilage in the wound healing process is explained in Figure 4.

### 7.4. Application of Mucilage as an Emulsifying or Suspending Agent

Mucilage can act as a good emulsifying and suspending agent. An emulsion is a thermodynamically unstable, biphasic system, containing two non-miscible liquids that can be classified based on their droplet size into several categories, such as nano-emulsions (20–200 nm), microemulsions (100 Å–100 nm), and macroemulsions (1–100 μm); therefore, they are extensively used in various industrial applications such as in the food and pharmaceutical industries [107]. Moreover, plant-derived natural emulsifying agents have effective emulsifying properties by imparting a charge to dispersed droplets, forming multimolecular sheaths around emulsion droplets by increasing the viscosity of a material [49]. The emulsifying properties of *Cucumis sativus* Linnaeus mucilage were also compared with commercially available tragacanth and gelatin with an additional valuation of their combined effect on the emulsion properties. They observed a high emulsifying capacity of *Cucumis sativus Linnaeus* mucilage as a primary emulsifying agent for oil–water emulsions. Likewise, Campos et al. [108] used chia seed mucilage as a replacer emulsifier for the formation of ice cream. Chia seed mucilage maintained the quality of the product, but its sensory properties were affected due to the dark color of mucilage. Moreover, plant-derived mucilage can increase the tensile strength of the hydration layer formed across the suspended solid particles by molecular interactions and hydrogen bonding. Therefore, these suspending agents perform well in the presence of moistening agents, as they do not reduce the interfacial surface tension. Thus, natural polysaccharides (mucilage) are commonly used as protective thickeners or colloids

### 7.5. Application of Mucilage in Tablet Formations

Mucilage is widely used for the preparation of tablets due to its high adhesive property, good swelling, and water absorption ability, therefore it can be used as a disintegrant or binding agent. Moreover, mucilage obtained from the seeds and fruits is used to sustain or modify drug release [109,110]. Mucilage is a binding agent that is pharmaceutical excipients used in the manufacturing of tablets to affect the coherence and aggregation of the mixed powder for the enhanced flow properties of particles and to impart physical strength to the tablet. Chaudhary et al. [111] manufactured tablets by using *Grewia asiatica* mucilage as a binding agent. During the in vitro dissolution study, released more than 80% of the drug within 30 min. They observed that percentage of friability and hardness of tablets was decreased with the increasing concentration of mucilage. 

### 7.6. Application of Mucilage for Removal of Contaminants from Water

Nowadays, plant-derived polymers (gums and mucilage) have attained a high demand in water purification due to their high water absorption capacities. Many components are responsible for water impurities, such as inorganic impurities (dyes), organic impurities (microorganisms), organic impurities (microorganisms), dissolved salts, heavy metals, and oils. moreover, seed or fruit mucilage is most effectively used towards such kind of water contaminators [112,113]. Furthermore, a Large concentration of contaminants in groundwater continues to be recorded as threats to the wellbeing of millions of people worldwide. Heavy metals are typically found in trace quantities in natural waters, but most are toxic even at low concentrations. Metals such as arsenic, lead, cadmium, nickel, mercury, and limited concentrations of zinc are highly dangerous for human health [114]. Heavy metals may reach the human body through food, water, air, or absorption through the skin when they come into contact with humans in agriculture, manufacturing, medicinal, industrial, or residential conditions [115]. Jones et al. [116] removed the selected heavy metal ions from the aqueous solution by using mucilage of *Dicerocaryum eriocarpum* (act as a biosorption). Mucilage was modified with potassium chlorides and sodium. Also Compared the potassium chlorides mucilage and sodium mucilage with non-modified deionized water mucilage. They observed that potassium chlorides mucilage increased the intensity of mucilage but did not produce new functional groups. The result of the study showed that *Dicerocaryum eriocarpum* mucilage has excellent binding attractions with heavy metal ions such as Fe, Zn, Cr, and Cd in the aqueous solution. In another study, Fox et al. [117] had worked on removing heavy metal from water using *cactus* mucilage. Intially, they extracted mucilage from (cactus) for the removal of arsenic. and prepared two types of the extracts (i) gelling extract and (ii) non-gelling extract. Their work mainly revealed an interaction of cactus mucilage with arsenic. -CO groups (carboxyl and carbonyl groups) and -OH (hydroxyl) functional groups interaction with the arsenate. The outcome from their work comes is that the gelling extract shows a better average than the non-gelling extract. Mucilage extracted from *Hibiscus esculentus* (okra) and *Malvasylvestris* (mallow) and have been successful in eliminating turbidity from wastewater due to their substantial flocculation activity, however, there has been an increased (COD) chemical oxygen demand [118]. consequently, *Viciafaba* and *Ficus-indica* mucilage are environmentally friendly bio-flocculants in the treatment of COD as well as in the decoloration and turbidity from textile wastewater and have shown the capacity for more contamination removal relative to industrial flocculants such as Polyacrylamide A100PWG and EPENWATE EXP31/1, and are more likely to be environmentally safe [119].

## 8. Therapeutic Importance of Mucilage

For many decades medicinal herbs and plants are used as traditional medicine for medical treatments. It is estimated that in developing countries around 80% of the people are still using traditional medicines for the prevention of diseases and among them, around 25% of drugs are derived from plants [7]. Many studies revealed that plant-derived mucilage such as (fenugreek gum, yellow mustard mucilage, and flaxseed) has many health beneficial properties due to their potential of reducing or preventing the risk of type 2 diabetes [67]. Mucilage is conventionally used in the medical field via topical or oral routes for urinary, respiratory, gastrointestinal, reproductive, and musculoskeletal systems and is also used for skin disorders. Moreover, they are also extending to diabetes, cancer, immunity stimulation, and have antioxidant, and antimicrobial property. Furthermore, mucilage containing plants such as *Trigonella foenum* graecum has antioxidative, antimicrobial, and anti-inflammatory properties [67].

## 9. Mucilage Based Nanocarriers and Their Application 

**Figure 5:**
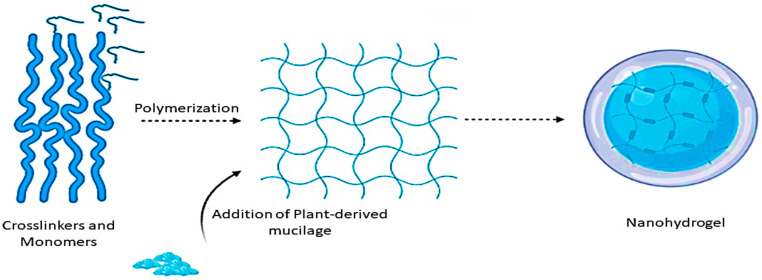
Synthesis of nanohydrogel using plant-based mucilage as an effective biopolymer.

Nowadays, synthetic and non-synthetic polymers have been successfully used for the formation of a hydrogel, but plant-derived (synthetic) polymers such as proteins, polysaccharides, and polypeptides are the most preferable choice, because of their extensive use of applications. Mucilage has an excellent potential to synthesize hydrogels because of its hydrophilicity, safety, and biodegradability. Hydrogels are hydrophilic and polymeric 3D material, which retains diffusive transport of liquids as well as also retains cohesive property of solids. They have attained high demand for technologists and researchers due to their extensive range of applications. The first synthetic hydrogel was prepared in 1960. Moreover, hydrogels from the plant-derived polymers are in high demand due to the presence of functional groups such as sulfate, amide, hydroxyl, and carboxylic which increases their swelling and water holding capacity, they are also interconnected with elasticity, and microscopic pores. There are many stimuli factors such as (pH, temperature, and electric field) [120]. There are generally two methods (physical and chemical crosslinking) that are used for the formation of hydrogel along with the principles of crosslinking of a polymer chain. The chemical crosslinking method includes the creation of new covalent bonds with the hydrogel’s polymer chain, while physical interaction can be also present between the polymer chain of the hydrogel. Both chemical and physical crosslinking methods can be applied for the synthesize of hydrogel from the plant-derived polymers (gum and mucilage) [121]. The formation of a nanohydrogel is explained in Figure 5. These characteristics increase the value of hydrogel as an applicant in food, pharma, and several industries [122].

The characterization of hydrogels is dependent upon the cross-linking (physical or chemical) measures during the formulation of the gel. Nanohydrogel is mostly similar to a normal hydrogel, which can be defined as a three-dimensional network of hydrophilic material (e.g., polysaccharide) with a diameter of less than 100 nm. Nanoparticulates have many benefits when compared to micro and macrocategorization in food and several other industries. The term nanohydrogel was first introduced to describe the cross-linking and networking of poly-anions [123]. They are used in several applications such as wound healing, drug delivery, vaccine delivery, the enhancement of film properties, and enzyme immobilization [124,125]. Mucilage-based hydrogels containing nanocomposites form a 3D network of extreme porosity, which allows a large absorption of food or drugs in water [126]. Nanocomposites are divided into three classes: ceramic matrix nanocomposites, polymer matrix nanocomposites, and metal matrix nanocomposites. They are chosen related to macro and microcomposites due to their excellent potential properties such as mechanical, barrier, and optical characteristics. The characterization of mucilage-based nanohydrogel can be performed through different methods such as field emission scanning electron microscope (FESEM), Fourier-transform infrared spectroscopy (FTIR), X-ray diffraction (XRD), X-ray photoelectron spectroscopy (XPS), and high-resolution transmission electron microscopy (HRTEM) [127]. The mucilage-based hydrogels can act as a protector, which prevents active ingredients from degradation, oxidation, and destruction, and also has several applications in water purification, drug delivery, the food industry, tissue engineering, and agriculture. Nanomaterials improve the barrier and mechanical aspects of food packages, and other developments for intelligent and active applications in the food industry [91]. Polymers containing hydrophilic groups such as -COOH, -OH, -CONH-, -SO_3_H, and -CONH_2_ interact with each other. Nanohydrogels are responsible for several stimuli such as temperature, electromagnetic field, pH, ionic strength, and light. Moreover, mucilage-based nanohydrogel is mostly utilized for the preparation of the edible coatings of edible films, and it is estimated that high mucilage-containing seeds or fruits are a good source of edible gum and can be used for various applications [122]. Nanohydrogels combine the great characteristics of hydrogels, such as absorption capacity, hydrophilicity, flexibility, great water holding capacity, with the advantages of nanoparticles, allowing for obtaining better dispersion in food packaging material and decreasing the number of bioactive compounds to be applied [128]. Plant-derived mucilage-based nanohydrogels are in great demand due to their unique properties, such as biocompatibility, biodegradability, stimuli-responsive properties, and biological characteristics, making them a good material for selection in diverse applications. Furthermore, nanohydrogels have potential applications such as controlled drug delivery, biomimetic materials, and biological or chemical sensors. Nowadays, nanotechnology plays a very important role in drug delivery systems, food applications, and water purification [129]. Therefore, nanoparticles (magnetic and non-magnetic), nanofibers, nanocomposites, and nanoencapsulation are widely used as nanocarriers in various industrial applications, such as for the controlled delivery of drugs, the removal of dye, and the development of film, which are highlighted in Table 5.

Moreover, Rayegan et al. [130] synthesized magnetic Fe_3_O_4_ nanoparticles coated with basil seed mucilage for the application of the controlled drug delivery of an antibiotic (cephalexin). The sample was characterized using XRD, FTIR, TEM, FESEM, and VSM. One-hundred and fifty magnetic nanoparticles were randomly selected for FESEM, which showed that the mean size of the nanoparticles was 6 nm and 12 nm, with 0.25 and 0.28 PDI values, respectively. Moreover, the antibacterial efficacy was evaluated by the disk diffusion method, and it was observed that there were no negative effects on the performance of drugs or on the structure by the loading of cephalexin onto the basil seed mucilage-coated magnetic nanoparticles. Moreover, it also increased the antibacterial properties of cephalexin. Consequently, Mohammadi et al. [91] prepared nanocomposite films based on okra mucilage (OM), carboxymethylcellulose (CMC), and ZnO nanoparticles, and evaluated their antibacterial and physicomechanical properties. In their study, they used different proportions of okra mucilage and carboxymethylcellulose (0/100, 30/70, 40/60, and 50/50, respectively). Colored films were observed with high levels of ZnO nanoparticles and okra mucilage. Moreover, due to the addition of mucilage, tensile strength was increased and elongation at the break value was decreased by the incorporation of ZnO nanoparticles into carboxy methylcellulose film.

### Synthesis of Nanoparticles with Mucilage

Nanoparticles have attained widespread attention for the development of green, facile, and sustainable synthesis of nanoparticles. Physicochemical methods such as direct precipitation, microwave, and hydrothermal methods are widely used [132,133]. Therefore, plant-derived polymers such as gums and mucilages are effectively used for the preparation and functionalization of nanoparticles. Mucilage used for the formulation of microparticles was also exploited in nano-formulations using vitamin D loaded nanoparticles on cress seed mucilage and gelatin. The prepared particles enhanced encapsulation efficiency (around 70%), with better in vitro release percentages of vitamin D in intestinal fluids and simulated gastroitestinal conditions [130]. Consequently, a study conducted by Pathak et al. [134] explained the achievement of using mucilage as an anionic ocular polymer for the synthesis of nanoparticles with a cationic polysaccharide polymer (chitosan). The antibacterial activity of the prepared sample was investigated against Gram-positive (*Bacillus cereus* and *Staphylococcus aureus*) and Gram-negative bacteria (*Salmonella typhimurium* and *Escherichia coli*). This type of nanoparticle can be useful in the medicinal industry for sustaining the anti-bacterial effect of antibiotics. Furthermore, the negative charge of the mucilage was additionally subjugated through the preparation of cephalexin-loaded/basil seed mucilage-coated iron oxide (Fe_3_O_4_) magnetic nanoparticles. The in vitro release of antibiotic (cephalexin) from the drug-loaded coated nanoparticles at pH 7.4 displayed biphasic form, starting with an initial quick release, then a continuous release phase. Apart from abusing the negative charge of the mucilage, phase inversion techniques and anti-solvent supercritical gas using supercritical carbon dioxide as an anti-solvent were applied for the preparation of mucilage nanoparticles loaded with the cytotoxic drug paclitaxel. The nanoparticles showed high drug loading above 75, and small particle size (around 200 nm) [135]. Mucilage contains flavonoids and polyphenolic contents. These compounds can efficiently bind zinc ions from an aqueous medium, and they function as stabilizing agents and natural reducers during the synthesis of nanoparticles. During the oxidation process, several carbohydrates and the counter acetate ions are also decomposed to produce high amounts of carbonate ions (Ag, Zn, Cu, and Co) and carbon dioxide; therefore, the binders can be removed [136,137,138,139,140]. 

## 10. Market Outlook of Mucilage

Mucilage is a gelatinous component that contains a good amount of proteins and polysaccharides, which are produced by almost all plants and a few microorganisms. It is abundantly present in agar obtained from red algae, and also in flaxseeds, fenugreek, chia seeds, marshmallow roots, psyllium, nopales, and aloe. However, a significant amount of mucilage that is used in industries is not available, and statistical consumption of mucilage is not reported. Earlier, it was primarily used for therapeutic purposes; however, it now has a variety of applications in the food, cosmetics, and health care industries [141]. The American and Asian markets have excessive utilization of mucilage in the ink, glue, and adhesive industries. The global market for cosmetics is growing, and so the use of natural products is as well. Therefore, the demand for mucilage for cosmetics will also increase in the upcoming years. Mucilage is useful as a binding agent in medicine, and, due to its healing properties, is used in ointments, and its cryoprotective property is increasing demands in the pharmaceutical industry [142]. The American and European mucilage market is in demand and is expected to grow positively during the forecast period. In the food industry, there is a growing demand for processed foods that contain mucilage as a thickening, gel-forming, and emulsifying agent. It is also used as a stabilizer in the milk industry, such as in ice cream, yogurt, and, flavored milk. Moreover, mucilage-derived hydrocolloids have been used in the food industry to provide textural functionality, such as in fruit fillings, water binding in meat products, dairy-based beverages, desserts, and jams [143]. Mucilages contain different kinds of antioxidants that slow the aging process, and thus can also be used in cosmetics products. Furthermore, mucilage is used for its health advantages, including bolstering the immune system, soothing the gastrointestinal tract, the ability to lower low-density lipids, and lowering blood pressure, which makes it applicable in pharmaceuticals and medicine. In the forecast period, there will be significant market growth in the pharmaceutical, health care, and medicine markets. 

Nowadays, intensive research is underway on plant-derived mucilage-based edible coatings and films to enhance the shelf life of fruits, vegetables, meats, and fish products. Similarly, mucilage is being used to develop nanofibers, which creates a large market for the textile industry in fabrication. The hydrophilic nature of mucus can act as a barrier to water penetration, delaying water loss and prolonging firmness [144]. This property makes it a promising component for cosmetic industry applications. Therefore, it is to be expected that, in the coming years, the slime market will include a wide range of applications and new market development.

## 11. Conclusions, Future Research Perspectives, and Challenges

In recent years, plant-derived polymeric carbohydrates have acquired high demand from food industries due to their diverse applications, such as as film coatings, emulsifiers, binders, and gelling agents. Among all carbohydrate polymers, mucilage has been abundantly used in the field of modern science due to its diverse applications. Plant-derived mucilage can be obtained from the special mucilage cells of different plant parts. Moreover, mucilage is mainly composed of complex carbohydrate polymers with highly branched structures that consist of L-arabinose, D-xylose, D-galactose, L-rhamnose, and galacturonic acid. They also contain glycoproteins and different bioactive components such as tannins, alkaloids, and steroids. Due to these properties, plant-derived mucilages can be used as an active functional ingredient, emulsifier, surfactant, stabilizer, encapsulating material, or cross-linker; therefore, they could be used for the fabrication of different types of nanocarrier. Moreover, researchers revealed broad-spectrum applications of biopolymer fabricated nanocarriers, especially nanohydrogels and metal nanoparticles, in the fields of medical and food sciences. Mucilage is a plant-derived polymer, their availability varies based on seasonal and environmental conditions. Apart from agronomical variations, these variations may affect the quality and production of mucilages; however, the extraction and purification processes are very complicated. Inadequate removal of mucilage, physical damage to the seed, and morphological features of the plant parts containing mucilage may all affect mucilage yield and consistency, posing a serious challenge to associated costs and the potential for mass production. The toxicity of mucilages is determined by their chemical composition and mode of action in food systems. As a result, it is important to determine the toxicity level of mucilages, particularly the presence of heavy metals in different plant sources. The toxic effects of mucilages can be determined using the fixed-dose procedure, which is recommended by guideline number 425 of the Organization for Economic Cooperation and Development (OECD). Mucilage contains around 10% moisture content, therefore the risk of microbial contamination during any stage of their processing may be high. This is due to the presence of biological substances that promote the growth and production of microorganisms in favorable conditions. Furthermore, the storage period is also a key factor in the contamination of mucilage. Studies have reported that variation in storage conditions leads to changes in the quality of mucilage. This often requires proper control of the different handling methods used at various stages of the supply chain. Moreover, modifications can also be made to address the disadvantages of uncontrolled biodegradability, shear instability, thermal decomposition, pH dependence, thickening, and uncontrolled hydration.

## Figures and Tables

**Table 1:** Origin of mucilage in different parts of a plant.

Source of Mucilage	Part	Yield of Mucilage	Extraction Method	References
*Linum usitatissimum* L.	Seed	7.3%	Extracted by centrifugation	[28]
*Spinacia oleracea* L.	Leaves	12.62%	Extracted by acetone	[29]
*Salvia hispanica*	Seed	8.3% 6.4%	Non-thermal extraction Thermal extraction	[30]
*Plantago major*	Seed	15.18%	Thermal extraction	[31]
*Basella alba*	Leaves Stem	4.83% 6.20%	Hot water extraction	[32]
*Psyllium seed*	Husk	37–52%	Ultrasonic bath extraction	[33]

**Table 2:** Functional properties of various plant-based mucilage.

Mucilage Source	Functional Property	References
Chia seed mucilage	Stabilizing agent, thickening agent, fat replacer, and emulsifying agent.	[21,56]
Okra seed mucilage	Oil absorption, water absorption, emulsion stability, foaming capacity, and emulsifying capacity.	[65,66]
Tamarind seed mucilage	Water holding capacity, oil holding capacity, and solubility.	[13,67]
Flaxseed mucilage	Water holding capacity, thickening agent, gelling agent, emulsifying agent, and foaming agent.	[40,68,69]
Cress seed mucilage	water absorption, Film-forming agent, and gelling agent.	[70]

**Table 3:** Antimicrobial activity of different mucilage-containing plants.

Mucilage	Incorporated Agent	Gram-Positive Bacteria	Gram-Negative Bacteria	References
Basil seed mucilage	Zinc oxide nanocomposites crosslinking with borax	*Staphylococcus aureus*	*Escherichia coli*	[89]
Quince seed mucilage	Thyme essential oil	*Bacillus cereus*, *Listeria monocytogenes*	*Salmonella typhimurium, Escherichia coli, Vibrio cholera, Pseudomonas aeruginosa, Yersinia enterocolitica*	[80]
Chia seed mucilage	Oregano essential oils, cellulose nanofibers	*Staphylococcus aureus, Staphylococcus mutans, Bacillus thuringiensis*	*Escherichia coli, Pseudomonas aeruginosa, Salmonella typhmurium*	[37,90]
Okra mucilage	Zinc oxide nanoparticles and carboxymethylcellulose (CMC)	*Staphylococcus aureus*	*Escherichia coli*	[91]
Qodume shirazi seed mucilage	Lavender essential oil	*Staphylococcus aureus*	*Escherichia coli*	[92]
*Lepidium sativum* seed mucilage	Heracleum lasiopetalum essential oil	*Staphylococcus aureus*	*Escherichia coli*	[93]

**Table 4:** Application of various plant-based Mucilage as a coating material in the food industry.

Source of Mucilage	Coating on Food	Reference
Aloe vera	Application on tomatoes	[98]
Aloe vera and Basil mucilage	Application on Apricots	[99]
Barbery fig mucilage	Application coating on kiwi slices	[100]
Cress mucilage	Application on fresh beef	[93]
Shahri Balangu seed mucilage with cumin essential oil	Application on beef slices	[39]
Hibiscus mucilage	Application on tomato	[101]
Flaxseed mucilage and xanthan gum	Application on cheddar cheese	[102]
Aloe vera gel	Application on apple slices	[103]

**Table 5:** Application of seed mucilage with various nanocarriers.

Seed Mucilage	Nanocarrier	Applications	References
Basil seed mucilage	Magnetic nanoparticles (Fe_3_O_4_)	Application for the controlled delivery of antibiotic (Cephalexin)	[130]
Cress seed mucilage	Nanofibers	Application for the delivery of vitamin A	[18]
Quince seed mucilage	Zinc oxide nanoparticles	Application for photocatalytic dye degradation	[131]
Quince seed mucilage	Magnetic nanocomposites	Application for removal of cationic dyes from the aqueous solutions	[132]
Basil seed mucilage	Zinc based magnetic bio nanocomposites	Application for removal of azo anionic and cationic dyes from the aqueous solutions	[133]
Okra seed mucilage	Zinc oxide nanoparticles	Application for nanocomposites-based films	[91]
Basil seed mucilage	ZnO nanocomposites	Application for wound healing	[68]
Chia seed mucilage	Nanoencapsulation	Application as wall material	[115]

## Data Availability

Data sharing is not applicable to this article.
